# Protection from annual flooding is correlated with increased cholera prevalence in Bangladesh: a zero-inflated regression analysis

**DOI:** 10.1186/1476-069X-9-13

**Published:** 2010-03-22

**Authors:** Margaret Carrel, Paul Voss, Peter K Streatfield, Mohammad Yunus, Michael Emch

**Affiliations:** 1 Department of Geography, CB3220, University of North Carolina-Chapel Hill, Chapel Hill, NC, 27599, USA; 2 Odum Institute for Research in Social Science, CB3355, University of North Carolina-Chapel Hill, Chapel Hill, NC, 27599, USA; 3 Health and Demographic Surveillance Unit, International Center for Diarrheal Disease Research, Bangladesh, Mohakhali, Dhaka 1212, Bangladesh

## Abstract

**Background:**

Alteration of natural or historical aquatic flows can have unintended consequences for regions where waterborne diseases are endemic and where the epidemiologic implications of such change are poorly understood. The implementation of flood protection measures for a portion of an intensely monitored population in Matlab, Bangladesh, allows us to examine whether cholera outcomes respond positively or negatively to measures designed to control river flooding.

**Methods:**

Using a zero inflated negative binomial model, we examine how selected covariates can simultaneously account for household clusters reporting no cholera from those with positive counts as well as distinguishing residential areas with low counts from areas with high cholera counts. Our goal is to examine how residence within or outside a flood protected area interacts with the probability of cholera presence and the effect of flood protection on the magnitude of cholera prevalence.

**Results:**

In Matlab, living in a household that is protected from annual monsoon flooding appears to have no significant effect on whether the household experiences cholera, net of other covariates. However, counter-intuitively, among households where cholera is reported, living within the flood protected region significantly increases the number of cholera cases.

**Conclusions:**

The construction of dams or other water impoundment strategies for economic or social motives can have profound and unanticipated consequences for waterborne disease. Our results indicate that the construction of a flood control structure in rural Bangladesh is correlated with an increase in cholera cases for residents protected from annual monsoon flooding. Such a finding requires attention from both the health community and from governments and non-governmental organizations involved in ongoing water management schemes.

## Background

Several empirical studies have examined the impact of water construction projects on communicable disease rates. With the exception of onchocerciasis, whose vector habitat is fast moving streams, the rates of nearly every disease whose host or vector depend on standing water sources increase when water resource management programs are implemented by governments or NGOs [1-8]. Hughes and Hunter (1970) argue that programs which alter the human environment result in the formation of new 'ecological contracts,' contracts that typically have hidden or unanticipated costs. As Sow et al. (2002) contend, "feasibility studies mainly emphasize the economic benefits rather than the environmental and health hazards of water-resources developments." Even if public health is given consideration in the planning stages of water resource projects, the true impact of changes to aquatic systems on human health may not be known for decades. Directly comparing pre- and post-development health data provides the clearest understanding of how changes in water supplies have affected the disease experience of local populations [4].

Since the 1950s, flood control and water management have been central issues for the Bangladeshi government (officially East Bengal prior to 1947 and East Pakistan from 1947 to 1971). Starting with the 20-year Water Master Plan in 1964, and continuing through today, heavy emphasis was placed on the construction of embankments around the country [9]. Initially, water management efforts were organizationally top-down; there was little or no public input on decisions that had a very tangible effect on the daily lives of Bangladeshis. Water plans since 1999, however, have involved greater public participation and worked to address issues such as gender equity, social justice and environmental awareness while still focusing primarily on increasing agricultural production [10]. Yet, there remains little consideration of the potential implications of such infrastructure investment for disease.

**Figure 1:**
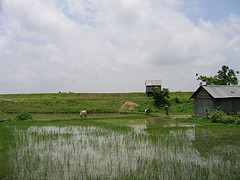
**View of the MDIP embankment from a bari located inside of the flood protected area**.

**Figure 2:**
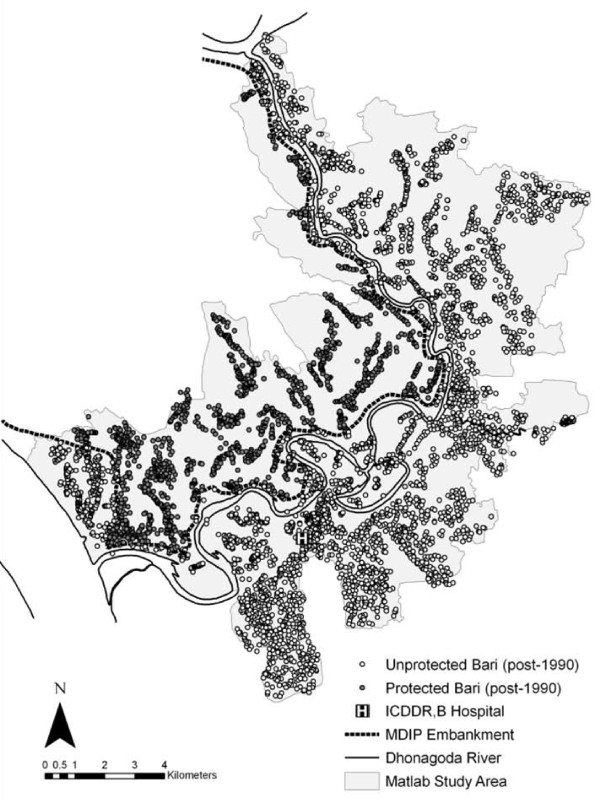
**Map of the main geographic features of Matlab, Bangladesh**. Baris protected from flooding by the MDIP embankment are darkened circles, unprotected baris are hollow.

Among the thousands of kilometers of embankments that have been constructed in Bangladesh is the Meghna-Dhonagoda Irrigation Project (MDIP), built in 1988-1989 and located in Matlab (Figure 1). The MDIP, completed between two particularly sizeable flood years for Bangladesh, consists of a 60 km ring embankment, irrigation and drainage canals, culverts, bridges and two pumping stations [11]. Matlab is a rural region, located ~50 km southeast of Dhaka where the Ganges and Meghna rivers join to form the Lower Meghna (Figure 2). Running from north to south through Matlab is the Dhonagoda River. The MDIP embankment along the Dhonagoda divides Matlab into two parts, one which experiences the seasonal flooding of the Dhonagoda and one which is typically protected. The protected area makes up about 40% of Matlab's 184 km^2 ^[2,12].

The population of Matlab is over 200,000 persons, with a population density of approximately 1000 persons per square kilometer [2]. The people of Matlab live within a structure of baris, or patrilineal household groupings. Since 1966 the population of Matlab has been under demographic and health surveillance by the International Centre for Diarrhoeal Disease Research, Bangladesh (ICDDR,B). Each resident of Matlab is assigned an identification code, as is their household, and twice a month each household is visited by a trained community worker who records demographic information such as births, deaths, marriages and illnesses [3]. Health information about the population of Matlab is gathered both at ICDDR,B's hospital and by the community health workers who visit each bari. Surveillance data shows that diarrheal and other infectious diseases are highly endemic to the population. One such disease, cholera, is highly correlated with annual flood events, with incidence peaking in September through December at the end of the monsoon season [13,14].

Thus, in Matlab there exists a unique opportunity to conduct a longitudinal analysis comparing disease pre- and post-implementation of a flood control program. In the present analysis we examine whether residence within the flood protected area of Matlab is associated with increased or decreased cholera prevalence. Previous studies have described a positive association between residence in the flood control area and cholera, but over much shorter time periods [3,12]. Analyzing two full decades of data for this area, including for years prior to the implementation of flood control and years following, provides a strong and robust test regarding the possibility of long-term implications for human health of flood control programs.

## Methods

Data from ICDDR,B's health and demographic surveillance system is joined to geographic information in a Geographic Information System (GIS) for Matlab. From the ICDDR,B health records, we have data on 8,500 laboratory-confirmed cholera cases between January 1, 1983 and December 31, 2003. This 21 year time frame spans the seven years prior to completion of the MDIP and the 14 years following. Cholera cases are assigned to the bari location where they occurred, and the total cholera count for Matlab's 7,490 baris is summed for each of the two time periods, 1983-1989 and 1990-2003. From ICDDR,B demographic surveillance we also calculate the mid-year bari population, and then sum the population counts for each bari across the 1983-1989 and 1990-2003 time periods to create a total background population at risk during each portion of the study period. Mid-year populations are appropriate to use for longitudinal studies such as this, since they approximately balance out the number of births and deaths that a bari experiences during each year. Summing these annual mid-year population counts also controls for the possibility of changing population sizes in baris across the duration of the study period, such that population growth or decline at the bari level is factored into individual baris' cholera risk. A dichotomous variable (0 indicating 1983-1989 and 1 indicating 1990-2003) is assigned to differentiate between the bari-level cholera and population counts for the pre- and post-flood protection period.

The original data set derived from ICDDR,B records had 14,980 entries: two for each of 7,490 baris, representing the cholera counts and population size before and after the flood control embankment project. We then removed baris with zero population counts in either period. Zero population records were more common in the first period because 1,609 baris were established only after 1989. This elimination process yielded a rectangular data set with 5,390 baris in each period.

The period cholera and population data for each bari are subsequently linked to a specific geographic location in the Matlab GIS. The GIS is accurate within 10 m and includes geographic features such as bari locations, the Dhonagoda River, the MDIP embankment and the location of the ICCDR,B hospital [2]. Using the GIS, each bari is assigned a flood protection status (1 for protected or 0 for unprotected). All records for baris in the 1983-1989 period are given a 0 flood protection status. Roughly one third of the 1990-2003 bari records are assigned a 1 flood protection status, indicating that they are located within the protection of the MDIP embankment.

Two environmental variables have been shown to be correlated with cholera in Matlab: distance to the ICDDR,B hospital and distance to the Dhonagoda River [2,3,12]. Straight line Euclidean distance between each bari and the nearest portion of the river and the hospital is calculated using GIS tools. These two variables are then added to the suite of bari-level information on cholera, population, period and flood protection status (Table 1).

**Table 1:** Model variables and their anticipated relationship to cholera prevalence.

Model Variables	Measure	Hypothesized Relationship
Period	1 (1983-1989)/0 (1990-2003)	Negative (due to overall decline in cholera incidence)

Background Population	Total count per period/1000	Positive (higher population increases cholera risk)

Flood Protection Status	1 (Protected)/0 (Unprotected)	Positive (being flood protected increases risk)

Distance to River	Euclidean distance (km)	Negative (due to decreased risk of flooding further from river)

Distance to Hospital	Euclidean distance (km)	Negative (due to distance decay of traveling to hospital)

		

Outcome Variable		

Cholera Prevalence	Total count per period	

Count data, such as the bari cholera counts examined in the present study, often are characterized by overdispersion and excess zeros [15-17]. Zero-inflated (ZI) regression is a practical way to model count data with both excess zeros and positive counts, as such models, incorporating covariates, can be estimated simultaneously in the extra zeros and the count distributional components of the model. Thus, ZI models are a highly useful approach to providing a parsimonious specification for non-negative integer-valued data with extra zeros, but only so when a key assumption underlying the models is plausible. These models assume that the data are a mixture of two separate data-generating processes: one generates only zeros; the other, usually a Poisson or negative binomial data-generating process, may also include zeros along with non-zero integer counts. Thus, the data contain more zeros than expected under standard Poisson, geometric or negative binomial distributions given the sample mean. Zero-inflated Poisson (ZIP) and zero-inflated negative binomial (ZINB) dual-state regression models have been widely applied in the social, economic, political and epidemiological sciences, although caution is warranted when the strong assumption of zeros arising from two processes cannot be sustained theoretically [18,19].

Baris in the Matlab study area reporting zero cholera cases are viewed as consisting of two subpopulations. The first subpopulation, producing only zeros, corresponds to baris that are free of cholera due to a presumed combination of positive local environmental or infrastructure factors (e.g., secure sanitary latrines, buried sewerage piping, uncontaminated ponds, and deep protected tubewells). Zero cholera counts for these baris may be considered "true" zeros. The second subpopulation consists of baris where cholera is actually prevalent but not reported, "false" zeroes. This situation can arise when an infected individual fails to seek medical assistance because, for example, the infection is relatively mild or when self-treatment with oral rehydration solution (ORS) is elected. A ZINB model can accommodate these excess "true" and "false" zeros, and also overdispersion (extra heterogeneity) among the positive outcomes that render a ZIP approach non-optimal. The parameter estimates in the count model, in our case a negative binomial formulation, test for correlation between variables and increasing counts. The zero-inflated parameter estimates, in contrast, represent correlation between the variables and a zero count. Thus, the parameter estimates for the count model and the zero-inflated models are typically of opposite signs.

**Figure 3:**
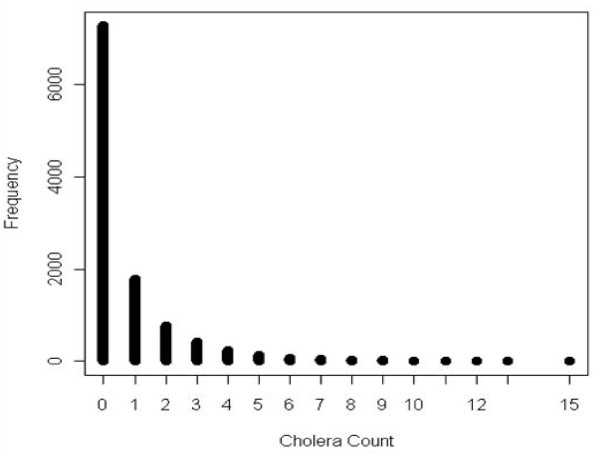
**Frequency distribution of the number of cases of cholera reported by individual baris in Matlab, 1983-2003**.

The distribution of cholera counts in the Matlab region during the period of study is overdispersed with respect to a Poisson distribution (empirical variance/mean = 2.881) and has an excess of zeros (70.2% of observations), suggesting the appropriateness of a ZINB regression model. The markedly skewed histogram in Figure 3 illustrates these features of the marginal distribution. Several alternative models were specified and estimated, including classical Poisson and negative binomial formulations, ZIP and ZINB models and so-called hurdle models which allow the probability of observing a zero to be independent of the mean number of counts but do not assume a dual-state data generating process [20]. Our final ZINB model (Table 2) was selected on the basis of strong goodness of fit and conformity to our theoretical sense of the underlying process generating the baris' cholera counts. Parameter estimation in the ZINB likelihood is carried out in R using the *pscl *package [21-24].

**Table 2:** Maximum likelihood parameter estimates of the multiple ZINB regression model.

	Negative Binomial Model					Zero-Inflated Model				
**Parameter**	**Estimate**	**S.E.**	**95% CI**			**Estimate**	**S.E.**	**95% CI**		

Intercept	0.476	0.050	0.377	0.574	***	1.708	0.128	1.458	1.958	***

Period (0, 1)	-0.315	0.045	-0.403	-0.227	***	0.891	0.158	0.582	1.200	***

FP status (0, 1)	0.535	0.047	0.442	0.628	***	-0.158	0.224	-0.598	0.281	

River distance	0.064	0.018	0.030	0.099	***	0.0990	0.071	-0.048	0.229	

Hospital distance	-0.222	0.007	-0.236	-0.209	***	-0.069	0.034	-0.137	-0.002	*

Population (1,000)	1.284	0.056	1.174	1.395	***	-13.029	0.962	-14.915	-11.143	***

log(theta)	0.418	0.059	0.302	0.534	***					

Significance values: 0 '***' 0.001 "**' 0.01 '*' 0.05 ' '

## Results

**Figure 4:**
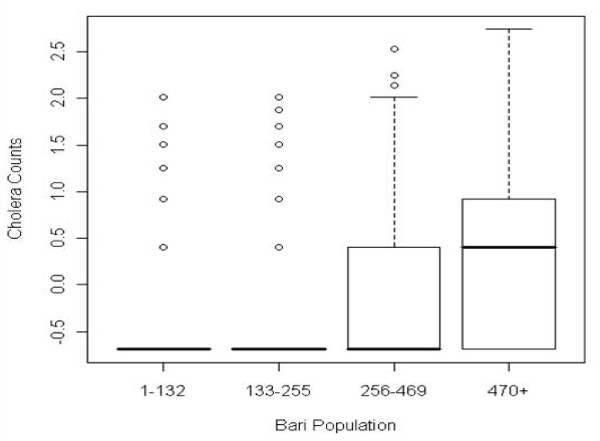
**Frequency distribution of cholera counts by bari population**. Baris with no population in either period have been removed from the analysis leaving a final N = 11,780 (5,390 baris, each represented twice in this graph).

The results of fitting the ZINB regression model to the Matlab cholera counts using the covariates discussed in Table 1 are shown in Table 2. Bari total population is included in the model to account for background population risk. Figure 4 reveals the anticipated relationship between cholera counts and population size, and this is reflected in the model parameter estimates as well. Population has a strongly significant control effect in both the count and zero-inflated part of the model. The very strong negative coefficient estimate in the ZI portion of the model implies that the larger the bari's population the lower the probability of it being cholera-free.

Cholera incidence is directly related to the river distance variable in the count model net of the other covariates. Cholera counts increase with increasing distance to the Dhonagoda. In the zero-inflation portion of the model the positive coefficient suggests, however, that as distance increases the frequency of zeros goes up, though this relationship is not statistically significant. These results suggest that the influence of proximity to the Dhonagoda River has complex interactions with cholera outcomes, as we explore in the following section.

The other distance variable in the model similarly behaves in an inconsistent fashion. Cholera counts decline with increasing distance to the ICDDR,B regional hospital. The most probable reason for this finding relates to difficulty of access, a consideration we develop more fully in the following Discussion section. However, the zero-inflation portion of the model further suggests that as distance to the regional hospital increases, the count of baris reporting no cholera cases decreases.

**Figure 5:**
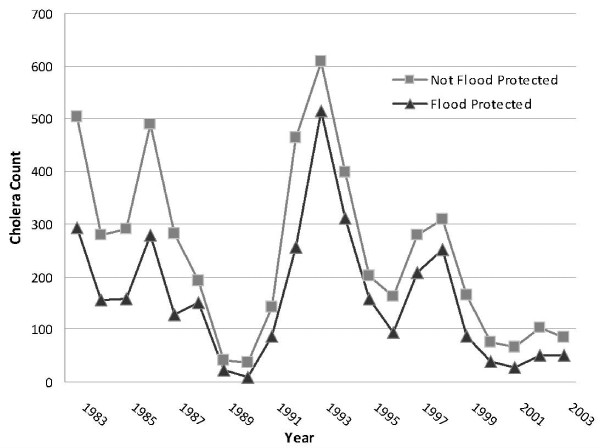
**Cholera counts in Matlab, Bangladesh from 1983-2003**. Cases are assigned to flood protection status based on bari of incidence. Cases taking place prior to 1990 are given the flood protection status of the bari following MDIP embankment construction.

The two key independent variables in this analysis provide the most interesting findings from the study. Cholera counts in Matlab declined overall in the second portion of the study period and the frequency of zero reports increased, net of the other covariates, as evidenced by the signs of the coefficients of the Period variable. We interpret this as a general decline in cholera incidence in the region after 1990 (Figure 5) and develop this thought in our Discussion section, below. What seems to be clear from the model is that this decrease following the construction of the Meghna-Dhonagoda Irrigation Project in the late 1980s is not responsible for the decline. Baris located within the flood controlled portion of our study area witnessed a strong and significant increase in the reporting of cholera following the erection of the flood control embankments and a positive flood protection status had no apparent influence on the number of baris determined to be cholera-free.

## Discussion

The background population of a bari is a large factor driving both cholera counts and the division between baris with cholera and those without. The total population of a bari represents the background risk, the number of opportunities for a cholera case to exist. Thus, as the population of a bari increases the number of potentially infected individuals increases. Among baris in the negative binomial count model, population is highly statistically significant and had the largest coefficient. For baris that experienced cholera, higher numbers of residents acted as a driver of higher cholera counts, there were more people be exposed and infected by the cholera bacteria. Among baris with no cholera, population had a strong negative effect, with a coefficient of -14.08, the largest in either model. As the population of a bari increases in the zero-inflation model, the chance of baris remaining cholera-free greatly decreases. Among baris in Matlab, the total population of a residence has the largest effect on both any cholera cases occurring and of higher numbers of cases.

The period variable (where 1 represents the 1990-2003 post-flood control period) was significantly associated both with baris that experienced cholera during the study period and with baris that did not, signaling a global decline of cholera in the study between the pre- and post-flood control infrastructure (Figure 5). Among baris with zero cholera counts, a positive association with period indicates that the 1990-2003 period is more strongly associated with an absence of cholera in a bari than is the 1983-1989 period. Cholera in Matlab is decreasing as general socioeconomic status improves, as access to clean water via tubewells increases, and as availability and knowledge of prevention and treatment strategies spread [25].

Although travel to and treatment at the ICDDR,B hospital is free for Matlab's residents, there still exists a distance decay effect across baris. Residents who live further away from the hospital, which is located in the southwestern portion of the study area (Figure 2), appear to be less likely to regularly report to the hospital and have cholera diagnosed. This distance effect is clearly seen in the negative relationship with hospital distance in the negative binomial count model, where baris distant from the hospital do experience cholera counts but in lower numbers than baris close to the hospital. Paradoxically, however, the weaker (negative) relationship in the ZI portion of the model suggests that as hospital distance increases the number of baris reported as cholera-free declines. While explanations for this relationship remain unclear, it might be due to underlying spatial patterns of unmeasured variables such as differing perceptions of risk associated with non-treatment of cholera-like symptoms or preference for self-medication with ORS.

The relationship between cholera and proximity to the Dhonagoda River is similarly paradoxical. Baris that are further from the river either reported more cholera cases or more frequently reported zero cholera. We had hypothesized that there would be a negative relationship between cholera and river proximity, believing that the flooding of the Dhonagoda River constituted a primary source of potentially cholera-contaminated water. Perhaps, however, the opposite is true, that baris distant from the river are more dependent on stagnant or backwater sources of drinking, cooking and bathing water, sources that might have higher levels of bacterial contamination. The river also acts as a primary transportation route through Matlab, so it could be that baris further from the river are less likely to travel to the hospital, and thus more frequently report zero cholera. While we are unsure of the precise relationship that our model indicates, it is clear that the Dhonagoda River has complex and spatially diverse effects on cholera outcomes.

Residence in the flood protected area of Matlab, when all the other environmental variables are controlled for, has a statistically significant positive effect in the cholera count model. It has no effect in the zero-inflation model. Thus, while the implementation of flood protection appears to have had no effect on whether a bari experiences cholera, it does act to increase the number of cholera cases among baris with cholera. While the positive relationship between flood protection and cholera may seem counterintuitive, it helps to think of monsoon flooding as a natural part of the aquatic cycle in Matlab. Monsoon flood waters act as a system reset, flushing out depleted and potentially cholera infected water supplies. We suggest that baris protected from this flooding have greater continued exposure to cholera bacteria in the environment, either through natural occurrence or through contamination of water supplies by human waste. Alternately, rather than an environmental explanation, we conjecture that there could be a behavioral change responsible for the relationship between flood protection and increased cholera counts: if people believe themselves to be better-protected from choleric water supplies they could relax their guard against interacting with contaminated water.

The process by which an individual resident of Matlab is exposed to and becomes infected with the cholera bacterium is dependent on the dynamic interactions of a wide array of population and environmental factors. Whether that individual goes on to infect others is further dependent on the timing and occurrence of these variables. While we have examined only a few of the many factors believed to be important to cholera outcomes, it is useful to examine the broad patterns by which the residential environment of Matlab contributes both to the presence of cholera in a bari and to the propagation of the disease among certain baris over time. Our results illustrate that many factors important to the degree to which a bari experiences cholera are also important in determining whether a bari experiences cholera at all. The alteration of Matlab's traditional aquatic cycle for a portion of the study population via the construction of the MDIP embankment, however, appears to act as a driver of higher cholera counts, and does not confer any significant protection against a bari ever experiencing cholera.

## Conclusions

Ongoing construction of flood protection structures in Bangladesh, and the continued alteration of water ecosystems for economic development in other countries, will likely continue to have adverse effects on human health until these mechanisms are better understood. While those in favor of water management schemes can list increased agricultural productivity and other benefits, the fact remains that the planning, implementation and monitoring stages of such projects need to explicitly account for increased occurrence of endemic waterborne diseases. Our longitudinal analysis of highly detailed and rigorously collected health data confirms previous studies that the introduction of flood protection in Matlab amplified cholera occurrence, even as the overall incidence of cholera in Matlab was on the decline. These unintended "side effects" must be considered by health officials in Matlab and elsewhere as water systems are modified. Such considerations, when jointly made by environmental, agricultural, development and public health officials, should help public agencies work together meet mutual goals, among them direct public health mitigation efforts.

## Abbreviations

FP: Flood Protection; GIS: Geographic Information System; ICDDR, B: International Centre for Diarrhoeal Disease Research, Bangladesh; MDIP: Meghna-Dhonagoda Irrigation Project; NGO: Non-Governmental Organization; ORS: Oral Rehydration Solution; ZI: Zero-Inflated; ZINB: Zero-Inflated Negative Binomial; ZIP: Zero-Inflated Poisson.

## Competing interests

The authors declare that they have no competing interests.

## Authors' contributions

MC conceived of the study, carried out the data preparation, participated in statistical analysis and drafted the manuscript. PV conducted statistical analysis and helped to draft the manuscript. PKS and MY oversaw the design and collection of the dataset and helped interpret findings in the context of the study area. ME conceived of the study and participated in its design and coordination and helped draft the manuscript. All authors read and approved the final manuscript.
